# Affecting photos: Photographs as shared, affective ethnographic spaces

**DOI:** 10.1177/14687941241246173

**Published:** 2024-04-22

**Authors:** Jennifer Rowsell

**Affiliations:** 7315 University of Sheffield, UK

**Keywords:** affect, family photos, figured worlds, sticky objects, multimodality, visual methods, ethnographic approach

## Abstract

Scholars point to the ubiquity of visual images in media and popular culture as driving striking developments in visual research over the past decade. Yet, with this popularity, there is less attention paid to affective, non-representational dimensions of visual images and specifically to the ways that photos animate and inform ethnographic fieldwork. The felt, sensory qualities photographs hold play a role in not only what gets documented, but also what photos produce as shared, felt objects that circulate during fieldwork. This article redresses a gap in qualitative research literature on the affective, embodied co-experiencing of visual methods that happens during fieldwork by spotlighting a research study on family photographs. In the article, I begin by defining affect, then I profile extant non-representational, affect-driven visual methods and discuss how matter invites affect, and then I spotlight a larger research study I was involved in on visualising the modern Canadian family. In the article, I offer insights that emerged from photo-sharing interviews which produced what I call in the article, affective figured worlds. Built on Holland's concept of ‘figured worlds’ coupled with Ahmed's notion of ‘sticky objects’, the article explores the notion of affective figured worlds to attune researchers to more of the non-representational methods in play during visual research.

… looking at photos, it really does bring me back, it brings me a little jolt of the emotion that was captured in the picture.

David, December 2016

See Figure 1 which is an affecting family photograph from my adolescent years.

## Introduction

During the pandemic my Godmother sent me an old family picture. The photo opens the article combined with a quote by an interviewee from a research study on family photos. The picture captures a moment, an affect, an embodied sense that I forgot about and that was rekindled when I held the picture in my hand. One foot in childhood, the other in adolescence, it reminds me of a confusing time as a tween/teen when I was figuring out who I wanted to be. I don’t remember this precise moment, but I do recognise that period of time. I am confident that you the reader of this article have experienced similar image affects looking at a family photo.

In this article, I explore the notion of photo affect jointly experienced between the researched and researcher as they share family photographs. It is an article about co-experiencing affect through photo-sharing during ethnographic research. In my discipline, literacy studies, there has been an affect turn (Leander and Boldt, 2013; Lewis and Tierney, 2013; Ehret, 2018), but I appreciate that in other disciplines in the arts, humanities, and social sciences, affect theory is very familiar ground in research. What is under-represented, certainly in literacy studies and potentially in other disciplines, is how affect comes to the foreground and encircles qualitative, ethnographic fieldwork during photo-sharing moments and how photos and visual methods more generally animate affect and generate insights. Co-experiencing photos is a relational act where an object, in this case photographs, changes fieldwork and by extension, changes the quality of interactions and findings that can be drawn in visual research. You will note that there are only two visual images in this article. Family pics discussed in the article are far too personal to display and ultimately, the article is about what cannot be seen and what is felt and sensed. As a result, the article foregrounds the non*-*representational, the ephemeral forces you experience during fieldwork and what these affective pulls do for qualitative researchers’ analytical gaze.

The notion of affective figured worlds helps me as a researcher find a way into what image-sharing can do and foster in qualitative research. The argument rests on in-the-moment becoming in and through images and in this way, it is less about family pics and more about the experiencing of them. A key probing question is: in what ways does the notion of affective figured worlds contribute to greater insights about photos as semiotic resources used, shared, and analysed during qualitative research? How does engaging with family pictures open analytical cracks for researchers to probe deeper into how people experience memories, lived experiences, and ways that this experiencing impacts the researcher and the researched? The article starts by situating affect and non-representational approaches to visual data; then it argues for the notion of tacit modalities and immanent methods; and finally moves onto the larger research study on family photographs.

## Situating affect

Research profiled in this article involved me as a researcher sitting next to a person or people who shared family stories through photographs. The act of passing and holding pics or of turning pages in a photo album enveloped research moments and the sharing was not one-sided with participants telling me about the image, but it was a mutual space to share similar moments, sentiments, and shared affect. Affect therefore is at the heart of the article and as such demands defining, framing, and situating.

Looking closely at family stories and histories told through images, I examine sharing practices that took place over photo albums and tablets that gave me a window into three different families. I could not have predicted the nature and properties of these affective intensities – they were immanent, preconscious (Massumi, 2007), and emerged during conversations. Affect sat at the surface of interview exchanges and without the image to invite this sharing, I would not have developed such rich appreciation and understanding about each family that I spent time with during the research. There are connections here to Baruch Spinoza and his theorising of the ways that one body acts on another. Spinoza (translated by Allison, 1987) wrote about affect as a site of emergence and potential between people. Spinozan affect invites perceptual and embodied actions produced as one body acts upon another. Spinoza said, ‘I shall consider human actions and appetites just as if it were a question of lines, planes, and bodies’ (Allison trans of Spinoza, 1987: 7). This rendering of affect was different philosophically and ontologically from what came before Spinoza because earlier philosophers tended to elide feelings and embodiment with spirituality and out of body experiences of affect connected with religion and ecclesiastical experiences.

Research reported in this article considers family events and memories that become clear through co-sharing practices by scrolling through family pics on a tablet or passing them and talking and affecting through them. Looking and listening during image-based research calls on thoughts and ideas as much as it calls on feelings. During the family pic research, affect circulated as an invisible force between me and participants as they shared intimate stories about their histories through a picture. It was never a one-sided dialogue. Whether spoken or felt, there were deeper understandings that images permitted and that produced what I call later affective figured worlds from the work of Holland et al. (1998).

### Situating affect in image-based research

There is substantial research and writings on photography and affect across disciplines in the social sciences and humanities, but as I said, less so in my discipline of literacy studies. Reflecting on iconic writings about the emotive and affective power of photographs, there are theorists like Susan Sontag who wrote extensively about images and affect – in particular reflecting on her photographs of everyday American lives (Sontag, 2008). Sontag insisted that photos come alive through social conditions and social relations (Sontag, 2008). In the reported research, family pics elicited social relations and our talk around family pics drew in relational maps of families, friends, events, etc. Photos distribute social relations across time and space. Gell (1998) talked about the notion of ‘distributed objects’ that follow a memory trail and historical trajectories and photographs as objects in homes and on display in albums distribute memories across time and space (Gell, 1998). My own findings during this research resonate with Gell's observation that photos act and animate social relations.

What I witnessed during fieldwork in homes are the ways that stories are told around and with photos and how these stories are not exhibited in the photos themselves, but instead told around them, exorcised during conversations about what images ignite. Other image-based researchers have foregrounded this such as Edwards’ important contribution to image affect and the material impact of photos in terms of what gets bundled up in the experiences of holding and sharing photos (Edwards, 2012). In ‘Objects of Affect: Photography Beyond the Image’ Edwards reflects,

The stories told with and around photographs, the image held in the hand, features delineated through the touch of the finger, an object passed around, a digital image printed and put in a frame and carefully placed, dusted, and cared for, are key registers through which photographic meanings are negotiated. (Edwards, 2012: 224)

Edwards here refers to terms related to experiencing photos in place and through relations and I found myself going back in time during the research when Debbie (a participant whom you will meet) walked me downstairs to her living room and gave me a tour of her family by talking through photos of her five children in ascending order going down the stairs. There is a sense across theorising of photos by Edwards (2012) and Sedgewick (2002) that photos dislodge emotions during social relations and I certainly experienced this during our larger research study and in subsequent research on the notion of tacit modalities (Abrams and Rowsell, 2019). Photos, particularly family photos, connect strongly to life and events lived and they are a pull into the past and the present and the living and the departed.

Edwards talks about ‘placing’ as key to experiencing photos and indeed Debbie placed each photo of her five children deliberately going down the stairs and slowly told me the story of each one; as Edwards claims,

First is the idea of “placing.” I use this term to mean the work of a photographic object in social space through which questions of materiality, adjacency, assemblage, and embodied relations frame the meaning of the image. Second, to consider the material conditions of photographs themselves, I consider the remediation and repurposing of photographic images: the material translation of a photograph from one kind of object to another, and from one purpose to another. (Edwards, 2012: 226)

Edwards describes what I experienced in homes when photos catalyse a set of relations between the individual and me as a remediation of memories, of people, and of times gone by. Rose (2010) interpreted the ways that people experience family pics as affording certain kinds of experiences and thoughts in a moment. The ways that family photo albums carry with them subjectivities enlivened by social relations. As Edwards articulates it, family pics move from neutral objects before sharing to tactile, sensory plural modes of experiences (Edwards, 2012: 228).

In a special issue on affect and the limits of photography (Cartwright and Wolfson, 2018), contributors foreground the perceptual affordances and affective work that happens with photos and in particular, family photos. In the special issue, Phu and Brown (2018) profile their research on the *Family Camera Network*, which is a research study where researchers engaged in oral histories with participants who shared stories through family pics. The research examines a mutual constitution of affect through photographs and oral histories. What I found particularly insightful about Phu and Brown's research is how they identified both positive and negative emotions that emerge from family photos interfacing with oral histories. Phu and Brown showed how affect was on the edges of oral accounts and it was through the combined forces of image and words that sensory perceptions were triggered such as sounds, accents, and voices that played a role in the co-experiencing of images. During photo interviews, I too observed the ways that other senses were ignited as we talked through their chosen photos – gestures, sometimes different languages (e.g. Spanish), facial expressions – and these sensory, felt aspects impacted our photo sharing.

### Situating affect in matter

Edensor writes about the affective qualities of materials and modal dimensions encountered and experienced in places during qualitative fieldwork. In his book on industrial ruins, Edensor (2005) documented affective, sensory, and embodied properties experienced during fieldwork walking around industrial ruins like abandoned factories and warehouses. In his book on the topic, Edensor writes about the ‘sensual immanence’ of his experiences in an old, abandoned warehouse and ways that ‘the experience of travelling through a ruin’ sparked sensations, recollections, and emotions (p. 10). Edensor's research helpfully points to the tacit, sensory qualities of researching across contexts anchored in place (with place playing an informing role in the perceptual experiencing). Imagine upside down cars in open warehouse spaces or walking through an abandoned factory with a dirty doll on the floor or a piece of wet clothing on the ground. Broken up bits of detritus spark senses and emplaced synaesthetic (i.e. one sense sparking the next) feelings for Edensor which he documents in his research through such thick descriptions as:

The textures and tactilities, smells, atmospheres and sounds of ruined spaces, together with the signs and objects they accommodate, can be empathetically conjured up by the visual material in the absence of any realistic way of conveying these sensations, other than through words and images. (Edensor, 2005: 16)

Edensor's (2005) writings speak of ‘the heightened consciousness of material textures of the city, and the sounds and tactile experiences they produce’ (p. 91). He talks about an immanent immersion in space that does not require a physical, direct gaze, it is something that is felt through scenes. It is a somatic sense of life which I too felt with participants involved in the family photo research. In some ways, with the years that have passed since conducting the research and with the pandemic, when I return to the interviews these same affective intensities (albeit to a lesser extent) are ignited.

In Edensor's (2012) article on Blackpool's annual illuminations event, he similarly moves into and inside of the embodied, sensory, somatic research on his attunement to affective intensities experienced during the annual Blackpool light show. Since the 1920s, every autumn, Blackpool puts on a light show with an eclectic mix of illuminations from neon, lasers, fibre optics, and LED lights. Strewn around the city of Blackpool, the light illuminations string together a theme. Edensor describes clusters of varied illuminated stories that create and elicit ‘affective atmospheres’. These affective atmospheres envelope the city every year. Edensor extends his account of his synaesthetic experiencing of illuminated stories lighting up the city into the history of Blackpool as a city that has struggled economically with a downturn in tourism. The lights and ethos of the city thereby blend together. He applies Böhme's work on sentient experiencing of spaces and places to conceptualise the multiplicities of atmospheres that he experienced and what he describes as the ‘melding of affect, emotion, and sensation’ (Edensor, 2012: 1103). The benefit of Böhme's theorising of affect is that it effectively shows how a dominant mode like light bathes an atmosphere in a thick fog of modal density. I am referring here to the suffusion of light that fills the city as a dominant and presiding mode in the place and space.

Important to Edensor and my own research is an account of how ethnographic case study participants experience artefacts not only across time and spaces, but also in the moment of reception and how tacit modalities which is a term that I have developed with Sandra Abrams (Abrams and Rowsell, 2019) play a role in their affective meaning making. These social, cultural, and behavioural dimensions of ethnography also include colours, moods, feelings, emotional vicissitudes, and the general ebb and flow of affect (Pink, 2011). The concept of tacit modalities helps me to capture how people experience matter in the moment, as well as what matter resurrects for one on embodied, sensory, and emotive levels. This all serves as a backdrop to this featured study on a co-experiencing of family pics.

## Ethnographic research on family photos

Research reported in this article started in the spring of 2014. I collaborated on a SSHRC1-funded research project led by Andrea Doucet, entitled, Making/Re-Making Families: A Visual, Longitudinal and Cross-Cultural exploration of Family Practices, Family Photographs, and Stories. The research project aimed to advance theoretical, empirical, methodological, and public understandings about 21st-century family practices in a diverse configuration of Canadian families. The project focused specifically on family forms that were and are on the rise in Canada: LGBTQ+, new immigrant families, single parent, and working-class families. Devised by Andrea Doucet based on her research in sociology on families and care, the project involved a series of interviews with diverse families in their homes to discuss digital and analogue family photographs in albums, frames, and on screens.

The research presented in this article is ethnographic in nature (Heath and Street, 2008). Ethnographies most often document and analyse places or groups (Desmond, 2014) and this ethnography falls into the latter focusing on three different family groups with differing compositions (a same-sex family; an immigrant family; and a large working-class family) who share a common family habit and commitment to family photographs. Ethnographies of groups are concerned with habits, beliefs, interactions, behaviours, and artefacts and people are grouped together based on a common, shared practices (Fine, 2007). The research design began with researcher photo conversations in phase 1 and then into phase 2 when we interviewed our respective families 2–3 times over the course of the 5 years of the research. Sometimes we partnered up and went to each other's families to share in the data collection. Andrea Doucet accompanied me on some family visits for this project. The ethnographic dimensions of the study looked closely at interactions between researchers and participants and at artefact-focused moments when family pics were at the centre of interviews (Rose, 2010).

There are ethnographies that take sensory perspectives on ethnographic fieldwork. Ethnographers like Sarah Pink (2009) have argued that embodiment, senses, and affect can be apprehended, documented and written about in scholarly work and these sentient, felt properties circulate during the act of conducting ethnographic fieldwork. Pink (2009, 2011) recognises that ethnography involves documenting senses in contexts when affective dimensions are layered in active, conscious ways. Such a framing of ethnography moves from distanced accounts of practices, people, places and the passage of time to engaging with cultures by being in them, walking alongside community members, and sensing what it is to experience a particular context and their repertoires of practice together.

Over the course of two to three visits with families, I got to know their rituals, ways of speaking and being, and developed a felt sense about their homes. The manner of sharing family pics varied from one family to the next. Some had piles of family albums dated and logged by key events and milestones. Others focused on a range of digital and analogue photos stacked up for us to look through together. And yet another family shared analogue and digital photographs that they brought with them from their home country combined with Facebook and Instagram posts.

Some interviews happened in home spaces whilst others happened in a coffee shop. After conducting two interviews each with the three participant families, the data got put aside as sometimes happens with research and it was only during the pandemic after moving countries and experiencing the isolation and quietness of COVID-19 that I returned to what families shared about their photographic lives and visualised stories.

### Research background

The research study involved two phases of data collection that took place from 2014 until 2017. For the first phase, we met to talk about our own family photos and we answered the following question prompts: Who is in the photo? What event is taking place in the photograph? Where did it take place? Is there a story behind the picture? Each of us brought two to four family pictures to the interview. By coincidence, Diane Collier and I shared very similar family photos of our grandparents and quirky family scenes that involved fishing, sitting on a dock and being by the sea. After talking about our photographs, we devised questions to ask our respective families at their two sets of interviews. In this way, engaging with family photos and interviewing each other about them naturally led into phase two when we interviewed families about their family pics.

Then, after meeting as a team with our family photos and talking about our photos and then separately having a photo interview with a colleague (in my case it was Diane Collier), we then recruited families. The two sets of family photo interviews were differentiated temporally: the first interview was devoted to images from the past and the second family interview focused on present-day photos. Once I met with my three families, we set dates in our diaries and they set the terms for sharing – photo albums, curated photos on a tablet, or a mix of paper and digital photos. All interviews happened in homes. We encouraged families to reminisce through photos – looking back in time moving gradually into the present. We agreed to select participants who were diverse in nature to reflect modern family configurations and to render family as dynamic, diverse and complex. As mentioned, I recruited three local families: a large working-class family, a same-sex family and a Brazilian family who had immigrated to the Niagara region region in Canada where the research is situated. Interviews were booked near the end of 2016 and we went to family homes for the first interviews and returned to homes for the second interview. Each interview took an hour and a half and for some interviews, we sat in the kitchen or living area with photo albums on tables and on our laps or held framed photos in our hands or peering over someone's shoulder to look at photos on phones. There were structured interview questions such as what do the photos tell us about your family? In what ways have your photos changed over years? Where do you keep your photos? Other questions considered both the content of the photograph and what these photos signal about family life. Interviews were then transcribed and read by the team and, as I said, time passed and I returned to them more recently to reflect upon them and their implications for research methods.

What I remember most from the family photos is being in home spaces and sensing domestic spaces as relational and as lived in and through. Linking back to Edensor's research, in each home from Debbie's farmhouse filled with chachka and photos; to the sparse and virtually empty nature of Fernando and his family's apartment; to the sleek design of David's home. These moments were less about documenting an image and its relations to the family member and far more about nostalgic and affectively charged exchanges that characterised the research experience.

### Three families

There was a stated research aim within the larger research study to examine different types of families so that we had a cross-section of families, not privileging a certain type of family. The first family interview involved Debbie^1^ who is mother to five grown children, eight grandchildren, and one great granddaughter. Debbie lives in Niagara outside of a small city on a farm that has been in the family for generations. Debbie opened her home to me and shared funny, sad, memorable family stories through photos in albums and in framed photos on walls. Married for many years, Debbie talked about the steady decline of her marriage, her ex-husband's alcoholism and the impact of it on her children and on her own mental health. She remarried and has been very happy since and the photographs threw into relief the change in the family when she married Peter. What was clear from our conversations is that Debbie is at the centre of the family network: hosting Christmas every year; sewing outfits for grandchildren; and, supporting her adult children as much as she can. Having been the main care-giver when her children were small, there were a lot of ups and downs and financial struggles over the years. I gained an inside view of their family through the picture-sharing and felt like I came to understand family histories and stories by the end of our second home interview.

The second family interview took place with David whose partner is Nathan and they have an adopted son named Liam. Our first interview took place in his home in the Niagara region and the second took place in a coffee shop. David was very organised for our interviews and his storying told through family photos followed from his own story with pictures from his childhood to his early days with Nathan and then the first few days with Liam after his birth. What sits beneath David's storying is an awareness of being gay in a traditional family that did not accept his sexuality, with the exception of his Grandmother whom he was very close to until she passed away. As with Debbie's story, I felt privileged to hear David, Nathan, and Liam's family stories told through David's careful, thoughtful curation of family pics.

The third family shared their immigrant experience moving from Brazil to Canada and being in Canada for little over a year. Fernando, Alison, Peter (aged 8 then), and Jenny (aged 12 then) came to Canada for what they expressed as ‘a better and safer life’. For the two interviews, Andrea and I went to their apartment where they shared photos from their Brazilian life and their relatively new Canadian life. Fernando and Alison spoke openly about how disillusioned and even worried they became with life in Brazil and how anxious and scared they were for their children and their futures. They talked about tremendous disparities between middle and upper class families in Brazil compared with families who lived in poverty and how endemic these disparities are within Brazilian life. Leaving high paying jobs, a house, and a large extended family in Brazil to come to Canada was not easy. I vividly remember Andrea and I going to their apartment on a cold evening and they did not have furniture and the children had few toys and technology, yet they were so enthusiastic and positive about their new lives. Fernando and Alison apologised for having so few physical photos and limited digital images for us, but they were able to string together all of the stories that led to their move to Canada.

## Applying affective figured worlds to research

To interpret interviews, I discovered I needed a way to capture the co-sharing that I witnessed. By co-sharing, I am referring to stories of trauma, joy, grief and traditions participants discussed, my own similar childhood recollections, and ultimately, the bond that developed between me and participants as family memories were shared. I turned to ways of framing it and saw Holland, Lachicotte, Skinner and Cain's book *Identity and Agency in Cultural Worlds* on my bookshelf. In their book, they explore ways that people define and enact their relations with society to project subjectivities through actions, spaces, habits and habitual conversations, turns of phrase. These practices and habits form a bounded space of belonging that the authors illustrate through their case studies that they call *figured worlds*. Bounded figured worlds range from Nepalese caste systems to Alcoholics Anonymous groups to female college students’ dating lives. The case studies are as different as you can imagine, yet what cuts across all of them are habits, practices, speech patterns habituated over time and place shaped to the contours of time, space and place. Figured worlds gather individuals with little in common but shared actions, experiences, and common senses of self. A figured world is an ‘as if’ realm people form collectively (Holland et al., 1998). People seek out figured worlds to forge a sense of belonging, a reminder about who they are, what they care about, and where they come from. People's identities are often entangled within multiple figured worlds. A figured world can shift and reshape over time, structured by practices such as stating the 12 Steps of Alcoholics Anonymous. Although a sense of a culture matters in defining figured worlds, they are equally about story and imagination as a part of being inside of a figured world. Figured worlds happen as a part of a social process. Thinking about the family photo research, recounting family stories that sit within, around, and extend from a pictured moment represent a figured world with practices, shared understandings and memories, particular cultures and ways of being.

Researchers attune to figured worlds through observations in a research site, but there is an argument that figured worlds can also be attuned to and discovered through photographs. To illustrate the ways that figured worlds help me to think through the interviews, I offer Debbie as an example. Debbie stands out because she was so open about her life as we sat in front of her many photo albums. During our first interview, Debbie shared a moment when one of her five children went through a tough patch as she described it fighting off tears. Her son struggled with addiction and mental health and her ex gave her no support. As we turned pages in her photo album, Debbie stopped at pictures of her present husband and what the photos meant to her. Debbie shared that her second, present husband stepped into the father role at exactly the right time to support her and her son. She shared the story passing me photos from Christmas and birthdays and pointing out the consistent presence of her life partner over the years. We circled back to this story during our second interview. Part of the sharing involved mental health and I talked about my own family, depression and what brings a family together. This specific moment in the research was imbued with affective belonging producing complex interplays of stories. What hit home as a researcher are the emotional worlds that photos hold and how these affective intensities change with time and age. They are the moments that you never forget as an ethnographer.

### Combining figured worlds with ordinary affect and sticky visuals

Figured worlds envelope the self and everything that encompasses a self (race, culture, social class, convictions, beliefs, bodies and so forth) which includes objects and spatial features that sit within figured worlds. Thinking back to Holland et al.'s (1998) book, what sits with me is the opening vignette that throws into relief caste systems in Nepal with Gyanumaya arriving at someone's home as a woman from a lower caste is scaling the house façade so that she did not enter the house from the front door (because she was prohibited from entering through the front door within caste rules). This example involves more than Gyanumaya as a Nepalese woman from a lower caste, it involves the front door, the house façade, the balcony, objects Gyunumaya encountered on her way into the house from the balcony. To analyse an experiencing of such objects I draw on Ahmed's theory of ‘sticky objects’ (Ahmed, 2014). Ahmed's framing of emotions with objects takes account of the aboutness of objects: ‘emotions are both about objects, which they hence shape, and are also shaped by contact with objects’ (Ahmed, 2014: 7). On one side, there are emotions, affect, and on another side memories, attachments, cultural rules which together come to bear on encounters. As Ahmed (2014) expresses it, ‘emotions accumulate over time, as a form of affective value’ (p. 11). With roots in feminist and queer theory, Ahmed sits alongside other theorists like Butler to argue that emotions are a form of cultural politics, which helps to interpret emotions and shape the surfaces of bodies.

It is within the stickiness of emotions that people move with objects, spaces and across time. What I observed over the course of several interviews with families is a surfacing of emotions through photo-sharing in home spaces. As Ahmed (2014) maintains, ‘what moves us, what makes us feel, is also which holds us in place’ (p. 13) and certainly within the reported research, emotions encircle many moments during interview conversations and image sharing. These sensations impress upon us as researchers in the moment of research. Objects like family photographs can be an axis for research encounters that researcher-researched share and experience. It is about the relations between objects that vibrate, and that animate bodies in research and from which emotions accrue. In the following sections, I present findings from interviews that I analysed through an affective figured world lens.

## Visualising families: Affect and affective figured worlds in family pics

Reflecting on the *Visualising Family* research, family pics materialise events, people, memories and historical narratives, but what was palpable during interviews were the emotions family pics enact and communicate. During the research, interview conversations focused less on visible moments within photos and much more on the senses, perceptions and feelings that they exorcise. Photos were experienced beyond sight and language and were of what Ehret (2018) describes as an ‘amodal feeling of something happening’ (p. 564). So it is in this section that I analyse family photo interviews identifying the following themes: attuning to holiday pics, affecting parental selves, and finding self and family.

### Attuning to holiday pics

During our first and second interviews Debbie talked about Christmas. There were a number of Christmas rituals within her family and she documented these practices visually and pictures became a way to maintain and celebrate family togetherness. When Debbie shared her family pics with me, our conversations pivoted less around the photographs and more around the stories that they sustained. In fact, I don’t remember any of the photos that Debbie shared and she focused less on a photo's materiality and far more on what pictures represented within her family's repertoire of Christmas rituals. Indeed, what sits with me are the Christmas rites, practices, and events, like this excerpt during our first family interview:

Christmas is a ritual in our home. These photos show it. Kirk, my youngest, was born on Christmas Day. I used to have the kids take the camera and come downstairs to see what Santa Claus brought them. So, it was a ritual you see. This is before everything was torn apart … This tree here was our Charlie Brown Christmas tree. Christmas is a big deal in our family, especially as the family gets bigger. Debbie, 8 November 2016 (first family interview)

Christmas in Debbie's home not only involved practices like her ex-husband and now current husband dressing as Santa Claus, but also family spaces such as moving from upstairs and filming going downstairs to see what Santa put under the tree. Debbie lives on the same farm that has been with her family for two generations. Although there have been renovations over the years, the basic layout of the house remains the same. The tree with presents underneath it is always downstairs, next to the fireplace. What intrigued me as I sat with Debbie for both interviews is that there are no Christmas photographs on walls. Instead, framed photographs on walls going downstairs and in the large downstairs living room display groups of family members together and key celebrations like Debbie's 60th birthday celebration with all of her children and grandchildren. Yet, it was obvious to me that Christmas represents their family togetherness. Being inside of the visual figured world of Debbie's family means being bound up with Christmas and photo-taking:

Christmas was the time when we took photos – Christmas and Easter. My Mom and Dad would take photos like this. My Grandma and Grandpa lived with us until my Grandpa died. Then my Grandma had a stroke and my Mom took care of her right through. Mom was a trooper, she was so good and always made Christmas special. Debbie, 11 April 2017 (second family interview)

Moving from one generation to the next, there is an emphasis on matriarchs supporting rituals and rites around Christmas from Debbie's Grandma, to Debbie's Mum, to Debbie, and now to her eldest daughter Megan. Equally, there are practices displayed and associated with Christmas like baking cookies, crafting and sewing gifts, and decorating rooms. The figured worlds in Debbie's home are materialised in the Christmas tree and Santa Claus outfit (which has become quite threadbare over the years), but also in immaterial features felt through stories within photos. Losing her Mum or marital troubles were mentioned as we looked through generations of Christmas pics. I shared similar Christmas stories with Debbie during both photo interviews and it was more of the smaller stories (Georgakopoulou, 2015) that bonded us as interviewer–interviewee. What struck me over both interviews was a consistency and fullness to Debbie's visual figured worlds around Christmas memories. That is, other than her ex, and introduction of her current husband Peter, there were few absences or changes in who is in the photos. People aged, hairstyles changed, and children grew, but over many years they were all there with their arms around Debbie in the same home, unlike Fernando and his family whose Christmas rituals and figured worlds changed dramatically with their move to Canada.

When Fernando and his family moved to Canada, it was a sudden decision and they left good jobs and a large extended family to start this new life. Much of the change had to do with material circumstances. By this I mean Fernando and his family could not bring much to Canada when they immigrated and also, their financial situations changed substantially because they left well paying positions in Brazil to take minimum wage positions in Canada. They did all of this for a better, safer life for their children. In this excerpt from our first family interview, Fernando talks about how Christmas pics throw into relief the differences between their two lives:

So, this is very nice. It is a picture of our first Christmas here. So you have here is that we were very lost and a family we met said, we have a surprise for you. Then they brought us a, they assembled a Christmas tree and said, *now* it feels like home. So, this is our first Christmas without family or relatives. And in Brazil, Christmas is a strong thing, it is summer, it is different than here. We spend all night with the family, it's a lot of people and we drink, dance, and laugh a lot. But that tree really helped us feel at home here. Fernando, 16 November 2016 (first family photo interview)

What the excerpt signals is how much the Christmas tree meant for the family who were missing home and the larger community they have in Brazil. For them, Christmas means parties, dancing, late nights and they were longing for these experiences. The tree moved them and shifted their experiencing of the holidays. Returning to the picture gave them a space to appreciate this and share it with me. To be honest, there was less co-experiencing with Fernando and his family and that is mostly due to fewer photos, fewer shared experiences, and differences in culture and language. Alison and Fernando simply did not have as many photos as Debbie because they could only bring a few things when they emigrated to Canada. But what their Christmas picture did reveal is the contrast between the abundance of family, space and material circumstances in Brazil compared with safety, newness and far more modest lives in Canada. The gifted Christmas tree seemed to embody for them the dramatic difference in their lives.

### Affecting parental selves

Over the course of photo interviews, all participants referred to pivotal parental moments when they felt their parental agency. Across photo-sharing conversations, there were parental epiphanies and I was invited to experience these epiphanies with them as they shared photos. It was particularly prominent with David's family when he discussed the first time their adopted son Liam had a high fever:

This photo is the first time Liam got sick. Because I remember after Nathan had taken this photo I woke up and he was just burning hot. And he was just so, like a very cuddly child, very loving and affectionate, always wanted to be held. And at this moment, he was even more cuddly than usual. His body radiated so much heat. It's a little bit scary as a parent. David, 13 December 2016 (first parent interview)

David showed me this picture of Liam as a baby which seemed like a typical smiling baby photo. To me, it was a picture of a cute baby, but for David it rekindled his first worried parent feeling – the felt, embodied sense of a sick child and parental lived anxiety. Commiserating, I shared my own affecting parenting moment when our daughter got her first cold at 6 weeks and how unnerving it is to feel the heat coming off of a baby. For David, the picture reminded him of immense responsibility you feel when you look at a baby who is very sick. In Edensor's article about illuminations, he underscores the radiating presence of senses triggered as he toured the lights. Though not as overt in nature, David had carefully chosen this particular photo of Liam as the moment when he fully realised that he is a parent.

Later during the same interview with David, he shared a very different moment with Liam years later when Nathan, Liam and David were in Paris for a summer vacation:

When I look at this photo, it just reminds me of those moments and those times where it's just Liam and me and we have these bonding experiences and rituals. I think that it is important for Liam to have two very different experiences with Nathan and me. David, 13 December 2016 (first family photo interview)

There is a strong sense of a parental figured world within the photo that typifies David and Liam's parental bond compared with Nathan and Liam's bond. David spoke at length about how he is the parent who plays, constructs and imagines with Liam whereas Nathan is more about being practical, life lessons and being out doing routines with Liam. Where David will play apps and games with Liam, Nathan will bake with Liam. These distinct parental selves are often visualised and the visuals are deceiving in that they do not necessarily show different parental styles, it is more subtle and implied within the photographs. When you enter their home space, they have a rotating digital photo album in their kitchen with family pics that mark everyday moments combined with key events like going to Liam's Grandparents’ home for the holidays. When David showed me the picture of Liam beside him in Paris, it reminds me of Edensor's drawing out of Böhme's (2013) contention that stage productions can set ‘the conditions in which the atmosphere appears’ (p. 4). Similarly, David's careful framing of Liam growing up are atmospheric spotlights shared with me through the telling and sharing.

Fernando's family pics recalled specific emotions and convictions that he remembers – particularly the move from Brazil to Canada. Take this excerpt from our second family photo interview with Fernando:

Here is another picture that shows our choice as parents. We have satisfaction for being here because Brazil is so dangerous and everything that we did not feel so comfortable there anymore and made this decision for the children. This is not the best apartment that we have had, but it is the freedom that we have here and we know if Peter asks to go for a bike ride, I can say ‘yes, why not, go.’ In Brazil, we would never agree. Fernando, 12 April 2016 (second family photo interview)

When I think back to the family interviews, there were pivotal moments when participants became particularly animated, and Fernando did when he and Alison talked about their decision to move to Canada. Although they had very few photos from their Brazilian life, their stories intimated a grander life and references to ‘not the best apartment’ and to having very few possessions in their home, reinforced the change in lifestyle for safety, security and a better future for Peter and Jenny. The co-experiencing of images with the whole family involved not only scrolling through a few photos on a tablet, but also Jenny and Peter moving around the living room, adding in their own reflections impromptu, smells from their kitchen as Alison cooked, and animation at particular moments. Fernando was most definitely the most animated about moving to Canada for a better future of their family. Thinking back to my definition of affect from Spinoza, the family pics and what they elicited were much less about what could be seen and much more about their palpable relief being in Niagara and setting Canadian roots. The best way that I can think of describing this research process is that each family stood apart, unique, and sensed through image sharing. It would not have been the same if family pictures were not the centre of conversations. Implicit to this contention, there was an equal reliance on words said and images felt during interviews and as a combined force – talk around family pics made the research more powerful, more illuminating. In sharing photos, we found a rhythm and figured world.

### Finding self and family

Part of the interest and energy that David put into our family photo research was his curation of photographs for our interviews. David went as far back as his own childhood, his coming-out story, his early days with Nathan, adopting their son together, and Liam growing up. Debbie and Fernando did some curation, but David stands out for his attuning to self and his family life through pictures. Take this excerpt from our first interview:

I wanted to capture a wide view of our family. And I really wanted to start with this photo of just Nathan and I. This was when we first started dating. We went on a Caribbean cruise which was quite fun. And this kind of the beginning of our relationship. This was right around the time when we were becoming more serious and you know had conversations about wanted to start a family and what that might look like. David, 13 December 2016 (first family photo interview)

The photo marks a moment in their relationship when they decided to have a family together and the pic indexes this experiencing of self and family. David was careful and methodical about choosing photos for our first and second interviews and strived to capture rites of passage from his childhood family all the way to his grown-up family. There are associations immanent within each photo such as the heat and sun of the cruise ship or his childhood photo that he shared and how much it reminded him of a picture on the box of one of Liam's childhood toys. Such attuning folds in objects, places and spaces that signify family and a collective for David.

During my conversations with Debbie, she made it clear that her sense of self was strongly tied to being a Mum. Her home is filled with posed, framed photographs of her children and grandchildren. She gave me a tour of all of the framed photos in her house and I remember vividly walking down the back staircase and above each stair was one of her five children. I recorded the conversation and I remember pausing at stair #3:

This is Christian, he is in the middle of the five. He's my rock. He phones me almost every day, see how I’m doing, takes me to different doctor's appointments, he's very special. And, yes, he's one in a million. He used to have longer hair. In his grad picture he had long hair. Debbie, 8 November 2016 (first family interview)

There was a performative feel to Debbie's attuning to self and family in that the photos played a minor role, but mostly the interview opened-up a space to share her thoughts and reflections about her different children and their place within the collective. Listening to Debbie's words, there is an accumulation of life experiences with Christian that points to him caring and nurturing Debbie. Perhaps there is a role reversal here with her experiencing of Christian as a form of mothering for her. This moment of feeling and accumulation of practices reinforces for her a figured world within the family of experiencing Christian's care over many years and how it steadies her. There are hints here too of what Ehret (2018) calls temporal textures as varying intensities of time registered on the body. All of the interviewees mentioned these kinds of singularities within family units through photographs. The temporal textures rippled through all interviews and they invite past–present–future time travel about families and ways that they change, yet stay the same.

## Conclusion: Stepping back as I move forward

It took me a long time to return to, analyse and write up these family interviews. In truth, I put off writing up my part of the larger research study. I am grateful to the Principal Investigator, Andrea Doucet, for allowing me to write them up my own way because in the end it has illuminated overarching findings about visual methods (for me as a researcher who has applied visual methods throughout my career). Ultimately, after working on revisions of the article, I recognise ways of developing affective figured worlds further into future research. I have always admired Dorothy Holland et al.’s (1998) work developing figured worlds, but I never really found a tangible and meaningful way of applying it in my own research and writing.

As a coda to the article, I want to contribute one more family photo. Over the past year my Mum's best friend who is 94 has regularly emailed me photographs. The image on the next page is one of her shared images. The image is what my Aunt, as I call her, saw at dusk dusk from her bedroom window. She said in her email: ‘The bed faces this window. I love lying and watching the sky. Day or night. At night, some light from the ground lighting the sky. I love it!’ She lives with her son and his family in Brooklyn, NY. To me, knowing her well, the picture is soaked through with image affect. To you the reader viewing it, it might seem like a grainy, mundane, dark image, not sure really but I would likely see that (See Figure 2 for an affect-charged photograph shared by my Mum's best friend).

**Figure 1.**
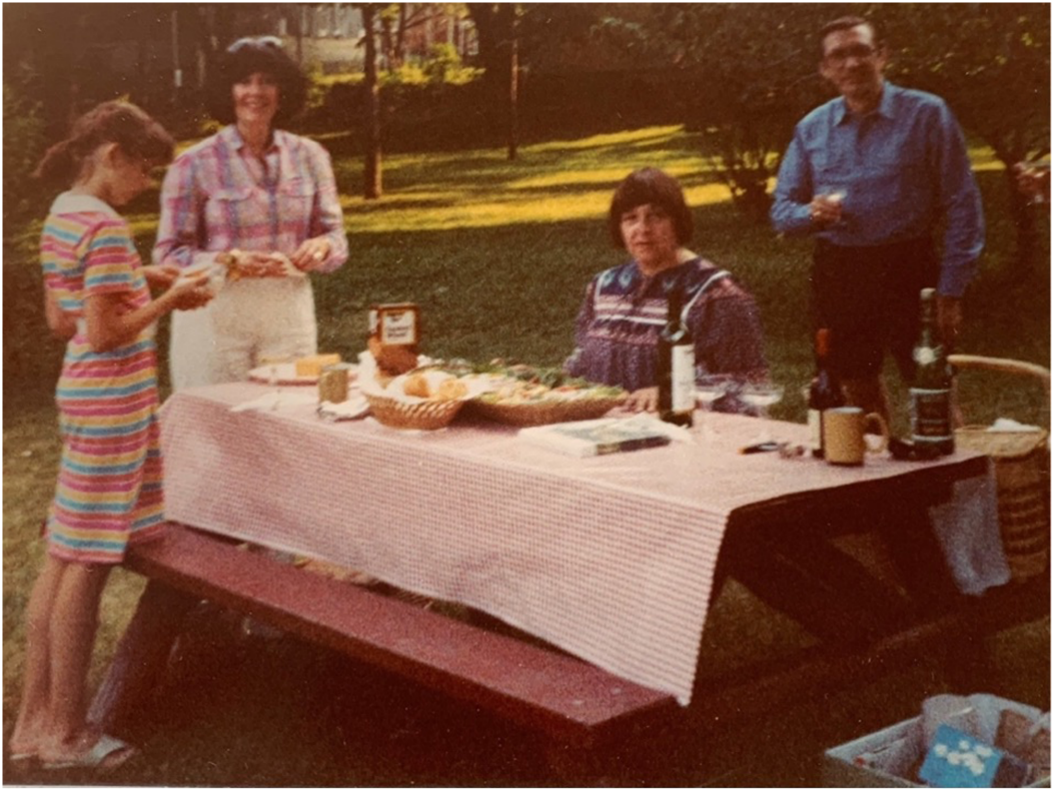
An affecting childhood photo.

**Figure 2.**
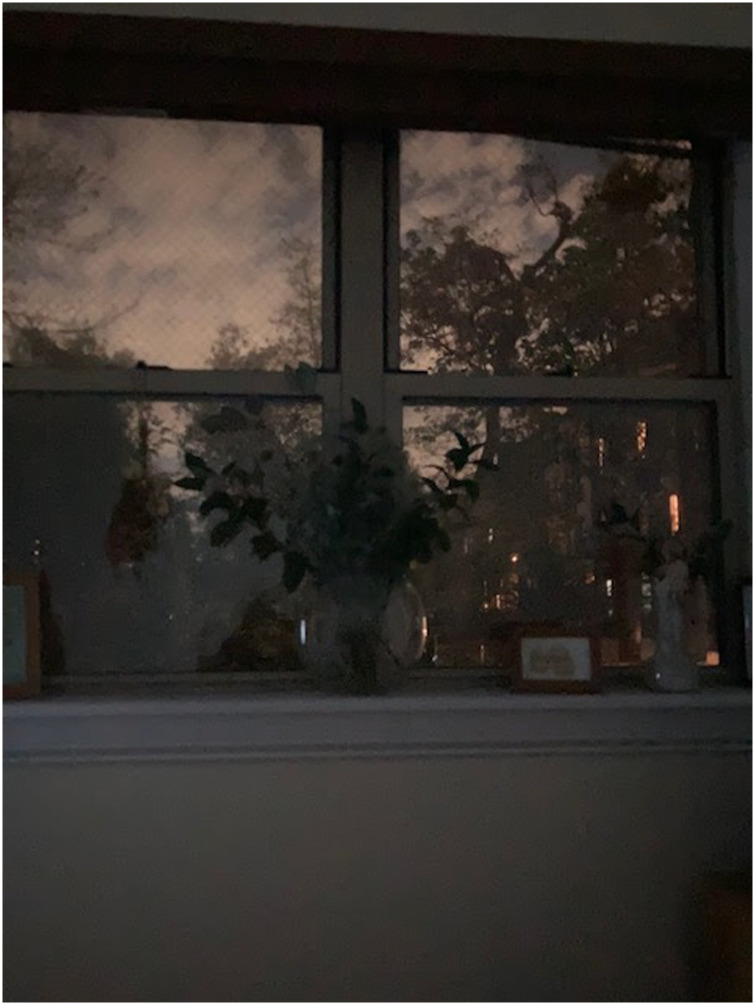
Light through my bedroom window at dusk.

The photo moves me because she shared it with me. It moves me because of the colour of the sky and what she sees and what I see. It moves me because it is sensed and not said. The gentle pinky-gray-brown sky, the barely decipherable objects on her shelf, and the vase with flowers – they are unassuming presences in her life that lift her spirits and give her pause to feel. Visualised and shared with me, I feel privileged to have a window into her affective world. It draws me into her every day and ultimately, in sharing the visuals, she shares her world with me. This image illustrates what Keane (2005) describes as ‘bundled signifying qualities’ (p. 183) which are visual properties for sure, but additionally, a co-presence of light, weight, gesture and feel that move people in all sorts ways.

Thinking back earlier in the article to extant literature on affect and image-based research, it was clear to me from the very first family interview that family photos are a platform to trace lives and to investigate ways that circuits of emotions travel across families – and I suppose my epiphany is how redolent they become when you hold a photo and talk about an event. Even as a bystander, I sensed Debbie's pain at bringing up five boys in an unhappy marriage and I could sense David's joy at starting a new family with Liam and Nathan. Family pics reconstructed senses, emotions, scenes and these research dialogues never would have taken place without handling and talking through images. What I regard as an original contribution to literature, or so I hope, is that these photo/object-centred moments qualitatively change a researcher-researched dynamic and relations and what is more and different is that the co-produced affect is what leads to understanding participants – their care; their stories; their familial compositions – in deeper ways.

Visual methods do this for researchers. Yes, visuals capture research cultures, participants, practices and actions and other visual researchers like Rose (2010) and Pink (2009) have similarly documented senses and affect experienced during fieldwork. But, what I hope that this article contributes is mattering affect that exists beyond words and language. That is, when language recedes to the background and other modes like visuals, sounds, gestures move forward, researcher perspectives change and what is significant is the sensed, felt and affected. Returning to the opening quotation by David, thinking about the research a few years on, I recall subtle jolts of emotion shared, experienced and made manifest in conversations. I only recall one or two photos like David's childhood photo and how much it looked like a boy on the front of a toy's packaging. I remember feeling lucky to hear about private family moments. I remember how voices changed when looking at a picture. I remember walking through homes or experiencing home spaces. These recollections became whole through a collective experiencing of families, what I have called an affective figured world in the article. I wrote this article in the hopes that it adds to growing movements in visual qualitative research to identify the unidentifiable and the felt in visuals. Diving deeper into felt relations circulating within and around family pics has analytical and ethical implications for research. Analytical in the ways that other researchers can interpret affective figured worlds within their visual research. Ethical in the ways that photos index far more than marking times and events and images carry with them the weight of emotions, feelings and senses that demand responsiveness yes, but responsibility too.

There is the photo that we see and talk about in research and then there are the stories, emotions, embodiments, beliefs and memories that are called forth but that are sometimes opaque during qualitative research. The trouble with affect and perception is that they are not reducible and, as a result, it is often difficult to capture them fairly and ethically in research accounts. I am humbled that Debbie, David, and Fernando and sometimes other family members met me on two separate occasions to talk through family pics. I am reminded of the important work by Rose (2010) on family photos and her own caution about respecting otherness when working with family photos; in her words, ‘that is, the specificity of those pictured should be recognised’ (p. 112). What seemed important was that participants chose photos and that they could ‘interrogate both how the other is being seen, and the grounds of possibility of that seeing’ (Rose, 2010: 113). A lingering question for me is, does the notion of affective figured worlds *other*? Possibly it does in some sense, but the driving force of approaching the data through affect and figured worlds is to capture how entangled the research became. From the research team meeting and sharing family pics; to asking ourselves the questions we asked of interviewees; to interviewees choosing pics; to meeting twice at a location of their choice about family pics over the years seemed to us ways of experiencing the research jointly. The fact that photos themselves did not feature prominently in the analysis, but instead the stories and worlds that they sustain is crucial to the research. This simple finding, that you do not need to show photos is important to me. So the way that I see it, there are two important implications for this kind of work: one considers the practice of photo-sharing and the other considers a shared experiencing of photo materialities.

For the first finding, what was distinctive across the families are sharing practices. There was a figuring out of stories through the back-and-forth of interview talk. Digital or analogue, family pics were never neutral and the telling of them became a key aspect of data collection. Debbie liked to walk around her home to perform framed photos. David strategically chose photos based on familial stages in his life. Fernando focused on contrastive photos of Brazil versus Canadian lives. What brought these practices together were affective figured worlds. The second finding considers the photo as an entity. There were certainly patterns that developed across family pictures dealing with events (weddings, Christmas and birthdays), emotions (joy, sadness and surprise), capturing individuality, capturing relationships, celebrating place and forging identities. Class, race, sexuality and nationality cut across family pics and this is not to say that experiences were not different based on these categories, but themes foregrounded in the article were common to all three families. Attending to what binds us within family photos through affective figured worlds seems to be a productive way for researchers to stem the tide of polarities to pull the researcher-researched binaries together.

## Notes

1 I use pseudonyms to protect the identity of research participants.
